# Stress, Allostatic Load, and Psychosis: One Step Forward in Research But Where to Go Next?

**DOI:** 10.3389/fpsyt.2019.00937

**Published:** 2020-01-09

**Authors:** Błażej Misiak

**Affiliations:** Department of Genetics, Wroclaw Medical University, Wroclaw, Poland

**Keywords:** schizophrenia, trauma, allostasis, glucocorticoid, hypothalamic-pituitary-adrenal axis

## Abstract

Stress exposure leads to the activation of several biological mechanisms that have been termed allostasis. These processes enable adaptation to novel situations; however; their prolonged activation exerts systemic and detrimental effects called the allostatic load (AL). The AL concept represents one of useful paradigms to describe biological consequences of chronic stress that might lead to a number of disease outcomes. The AL index, which is a collective measure of cardiovascular, metabolic, neuroendocrine, and immune dysregulations associated with stress exposure, has been found to predict morbidity and mortality in non-clinical populations. Consequently, it has been proposed that the AL concept might be a useful framework to describe biological consequences of chronic stress exposure in patients with psychotic disorders. This perspective article is an overview of studies investigating the AL index and its clinical correlates in patients with psychotic disorders. These studies have consistently reported elevated AL index in patients at the early and chronic course of psychosis. In addition, the AL index has been associated with a higher severity of positive and depressive symptoms, working memory impairments, and lower general functioning. The article provides some critical appraisal of studies in this field and indicates several future directions for investigating the AL concept in psychosis.

## Introduction

Psychotic disorders represent complex phenotypes with imprecise diagnostic boundaries. Prevalence of psychotic disorders has been estimated at around 3% and this group of mental disorders is perceived as one of most substantial causes of disability worldwide (1). There is a general consensus that the pathophysiology of psychosis is multidimensional with the involvement of several genetic factors, characterized by small effect size estimates, and environmental factors (2). Therefore, a paradigm shift toward implementation of models that might comprehensively capture biological alterations observed in psychosis is increasingly being recognized as a promising research perspective.

Accumulating evidence indicates that stress during critical windows of brain development might play an important role in the development of psychotic disorders (3). Approximately one third of patients with psychosis report a history of childhood trauma and this type of stressful experiences is a well-established risk factor of psychosis development (4, 5). Although exposure to early-life stress is neither necessary nor sufficient to cause psychosis, several studies indicate that a history of childhood adversities is related to a number of biological alterations observed in this group of patients [for review see (6)]. These alterations are represented by the hypothalamic-pituitary-adrenal (HPA) axis dysregulation, manifesting in elevated morning (7) and diurnal cortisol (8) levels, blunted cortisol awakening response (9), and attenuated cortisol response to stress (10). There are also studies showing lower levels of peripheral blood brain-derived neurotrophic factor (BDNF) (11), elevated levels of pro-inflammatory cytokines (12), more pronounced metabolic alterations (13), and altered DNA methylation profiles (14) in patients with psychosis and a history early-life traumatic stress. Patients with psychosis and self-reported childhood adversities present with unfavorable short- and long-term treatment and functional outcomes (15, 16). Finally, higher levels of stress biomarkers have been found to predict transition from ultra-high risk states to fully blown psychotic episode (17).

In light of several associations between stress exposure and biological alterations observed in psychosis, several models have been proposed to capture this complexity, including the reactive scope model (18), the adaptive calibration model (19), and the neural diathesis-stress model (20). Our group has also proposed that the allostatic load (AL) concept might be a useful paradigm since it provides a measurable construct (the AL index), summarizing various stress-related biological dysregulation (21). Afterwards, a number of studies investigated the AL index in patients with psychosis at various stages of illness. In this perspective article, I provide a brief overview of basic assumptions underlying the AL concept, review studies investigating the AL index in psychosis, and indicate future directions for studies investigating this paradigm in psychotic disorders.

## Definitions and a Brief Overview of the Allostatic Load Concept

Homeostasis alterations driven by exposure to stress lead to the activation of several biological processes that have been collectively termed “allostasis” (22). These processes enable adaptation to the demands of unexpected situations and act *via* a number of mediators, including i.e., hormones, neurotransmitters, neurotrophins, oxidative stress, and immune-inflammatory markers (21). The adaptive nature of allostatic processes is disrupted in case of chronic stress and continuous arousal. Indeed, prolonged activation of allostasis exerts systemic and detrimental effects. This condition has been defined as the AL (23), while diseases related to chronic stress and the AL have been termed the allostatic overload (24). The central nervous system gateways for mediators of allostasis are mainly represented by prefrontal cortex, the amygdala, and the hippocampus (25). The AL index represents a cumulative score of biological dysregulations measured by neuroendocrine, immune, metabolic, and cardiovascular markers. This measure has been found to predict mortality and morbidity, better than traditional detection methods, in several non-psychiatric populations (26). To date, several operationalizations have been proposed to calculate the AL index. However, a consensus statement regarding the calculation of the AL index has yet to be developed.

## Studies on the Allostatic Load Concept in Psychotic Disorders

A summary of studies investigating the AL index in patients with psychosis was provided in **Table 1**. To date, nine studies with overlapping samples of participants have investigated the AL index in patients with psychotic disorders (28, 32). Although various methods of calculating the AL index have been used, these studies have provided consistent evidence of elevated AL index in patients with schizophrenia (32–34) and first-episode psychosis (FEP) (27, 30, 31, 33). We also found similarly elevated AL index in patients with FEP and individuals at familial high risk of psychosis (FHR-P). The AL index has been found to decrease following antipsychotic treatment (27).

**Table 1:** A summary of studies investigating the allostatic load index in psychosis.

Study	Patients		Controls		Diagnoses	AL biomarkers	Clinical assessment tools	Main findings
	n (M/F)	Age (mean ± SD)	n (M/F)	Age (mean ± SD)				
Berger et al. (27)	FEP: 28 (19/9) SCZ: 28 (15/13)	FEP: 33.0 ± 11.5 SCZ: 40.1 ± 10.1	53 (36/17)	36.3 ± 11.5	SCZ and schizoaffective disorder	SBP, DBP, HR, BMI, WHR, creatine kinase, insulin, glucose, HbA1c, enRAGE, TG, TC, LDL, HDL, TNF-α, IL-6R, CRP, E-selectin, cortisol, metanephrine, normetanephrine, and copeptin	SCID, PANSS, and GAF	The AL index was significantly higher in FEP and SCZ patients compared to controls at baseline (with no significant differences between FEP and SCZ patients). The AL index was associated with significantly higher severity of positive symptoms (PANSS) and functional impairment (GAF, trend level significance). The AL index significantly decreased after 6 and 12 weeks of antipsychotic treatment.
Berger et al. (28)	106 (36/70)	17.2 ± 2.4	–	–	UHR	SBP, DBP, HR, cortisol, CRP, IL-6, IL-12, TC, TG, and BMI	CAARMS, BPRS, SANS, YMRS, MADRS, SOFAS, GF-R, GF-S, and CGI	Higher baseline AL was associated with poor social and occupational functioning (SOFAS) and more severe manic symptoms (YMRS) after 6 months of observation. No significant associations were found after 12 months.
Chiappelli et al. (29)	44 (28/16)	32.7 ± 12.6	33 (19/14)	35.3 ± 14.2	SCZ and schizoaffective disorder	SBP, DBP, HR, BMI, WHR, HDL, TC, HbA1c, CRP, urine epinephrine and norepinephrine, urine cortisol, and blood DHEA	SCID and BPRS	The AL index was significantly higher in the group of patients compared to controls. Whole brain average cortical thickness was significantly lower in the group of patients than in controls. Once the AL index was accounted, group differences in cortical thickness appeared to be insignificant. Elevated levels of CRP, stress hormones, and cardiovascular indices were related to cortical thickness in patients. In the group of controls, only CRP levels predicted cortical thickness.
Misiak et al. (30)	36 (20/16)	27.5 ± 7.4	31 (12/19)	25.2 ± 6.6	FEP: SCZ, schizophreniform disorder, brief psychotic disorder, schizoaffective disorder, and delusional disorder	SBP, DBP, BMI, WHR, CRP, fibrinogen, albumin, glucose, insulin, TC, LDL, HDL, TG, cortisol, and DHEA-S	OPCRIT, PANSS, SANS, SAPS, HDRS, YMRS, GAF, SOFAS, COPE, CISS, LTE, and PSS	The AL index was significantly higher in FEP patients compared to controls. Patients with FEP were less likely to use active and task-focused coping. Lower odds of using these coping styles, planning as well as positive reinterpretation, and growth were related to higher AL index in FEP patients, but not in controls. Depressive symptoms were associated with lower likelihood of using task-focused coping as well as positive reinterpretation and growth, and were related to higher AL index. Depressive symptoms mediated the effects of task-focused coping as well as positive reinterpretation and growth on the AL index.
Misiak et al. (31)	40 (21/19)	27.6 ± 7.4	35 (14/21)	25.5 ± 6.7	FEP: SCZ, schizophreniform disorder, brief psychotic disorder, schizoaffective disorder, and delusional disorder	SBP, DBP, BMI, WHR, CRP, fibrinogen, albumin, glucose, insulin, TC, LDL, HDL, TG, cortisol, and DHEA-S	OPCRIT, PANSS, SANS, SAPS, HDRS, YMRS, RBANS, GAF, SOFAS, LTE, and PSS	Elevated AL index was confirmed in FEP patients compared to controls. There was a significant negative correlation between the AL index and the digit span test score (working memory, RBANS) in the group of patients but not in controls.
Nugent et al. (32)	30 (17/13)	32.6 ± 12.1	20 (12/8)	35.7 ± 12.8	SCZ	SBP, DBP, HR, BMI, WHR, HDL, TC, HbA1c, CRP, urine epinephrine and norepinephrine, urine cortisol, and blood DHEA	SCID, BPRS, UPSA-2, and PSS	The AL index was significantly higher in patients. Higher AL index was associated with more severe positive symptoms (BPRS) and functional impairment (UPSA-2). Perceived stress (PSS) was not related to the AL index neither in patients nor in controls.
Piotrowski et al. (33)	42 FEP patients (21/21) 25 SCZ patients (14/11) 37 FHR-P individuals (12/25)	FEP patients: 27.7 ± 7.3 SCZ: 42.8 ± 13.8 FHR-P: 36.9 ± 11.0	42 (16/26)	27.8 ± 8.4	FEP, SCZ, and FHR-P (unaffected offspring of SCZ patients)	SBP, DBP, BMI, WHR, CRP, fibrinogen, albumin, glucose, insulin, TC, LDL, HDL, TG, cortisol, and DHEA-S	OPCRIT, PANSS, SANS, SAPS, HDRS, YMRS, RBANS, GAF, SOFAS, LTE, and PSS	The AL index was significantly higher in patients with psychosis and FHR-P individuals compared to HCs. Patients with FEP and FHR-P individuals had similar AL index. Moreover, the AL index was significantly higher in SCZ-AR patients compared to other groups of participants. Higher AL index was associated with more severe general psychopathology and depressive symptoms, lower scores of attention and semantic fluency, as well as worse general functioning in patients with psychosis. There was a significant negative correlation between the AL index and the scores of attention in FHR-P individuals.
Savransky et al. (34)	44 (28/16)	32.7 ± 12.6	33 (19/14)	35.3 ± 14.2	SCZ and schizoaffective disorder	SBP, DBP, HR, BMI, WHR, HDL, TC, HbA1c, CRP, urine epinephrine and norepinephrine, urine cortisol, and blood DHEA	SCID and BPRS	The AL index was significantly higher in the group of patients compared to controls. The AL index was significantly and inversely associated with fractional anisotropy of the fornix in both groups.
Savransky et al. (35)	58 (41/17)	36.1 ± 14.3	34 (20/14)	35.3 ± 14.0	SCZ and schizoaffective disorder	SBP, DBP, HR, BMI, WHR, HDL, TC, HbA1c, CRP, urine epinephrine and norepinephrine, urine cortisol, and blood DHEA	SCID and BPRS	The AL index was significantly higher in early psychosis patients and those with chronic illness compared to controls. Higher AL index was associated with more severe positive symptoms in patients with early psychosis.

AL, allostatic load; BPRS, the Brief Psychiatric Rating Scale (36), CAARMS, the Comprehensive Assessment of At-Risk Mental States (37); CGI, the Clinical Global Impression Scale (38); CISS, the Coping Inventory for Stressful Situations (39); CRP, C-reactive protein; DBP, diastolic blood pressure; DHEA, dehydroepiandrosterone; DHEA-S, dehydroepiandrosterone sulfate; FEP, first-episode psychosis; FHR-P, individuals at familial high risk of psychosis; GAF, the Global Assessment of Functioning (40); GF-R, the Global Functioning Role Scale (41); GF-S, the Global Functioning Social Scale (41); HbA1c, glycosylated hemoglobin; HDL, high-density lipoproteins; HDRS, the Hamilton Depression Rating Scale (42); IL, interleukin; LDL, low-density lipoproteins; LTE, the List of Threatening Experiences (43); OPCRIT, the Operational Criteria for Psychotic Illness Checklist (44); PANSS, the Positive and Negative Syndrome Scale (45); PSS, the Perceived Stress Scale (46); SANS, the Scale for the Assessment of Negative Symptoms (47); SAPS, the Scale for the Assessment of Positive Symptoms (47); SBP, systolic blood pressure; SCID, the Structured Clinical Interview for DSM-IV; SCZ, schizophrenia; SOFAS, the Social and Occupational Functioning Assessment Scale (48); TC, total cholesterol; TG, triglycerides; TNF-α, tumor necrosis factor-α; UHR, ultra-high risk of psychosis; WHR, waist-to-hip ratio; YMRS, the Young Mania Rating Scale (49).

Higher AL index has been associated with more severe positive symptoms and functional impairment in some (27, 32, 35) but not all studies (30, 31). In one study (28), baseline AL index was investigated with respect to clinical outcomes at 6 and 12 months of observation in individuals at ultra-high risk of psychosis. The authors also found a positive correlation between the AL index and a severity of functional impairment after 6 months. Additionally, higher AL index predicted higher scores of manic symptoms. Our group investigated the association between the AL index, depressive symptoms, and stress coping styles in FEP patients (30). We found that patients with FEP were less likely to use active and task-focused coping compared to controls. Lower odds of using these coping styles, planning as well as positive reinterpretation and growth were related to higher AL index in FEP patients, but not in controls. Depressive symptomatology was associated with lower odds of using task-focused coping as well as positive reinterpretation and growth, and was related to higher AL index. In addition, depressive symptoms mediated the effects of task-focused coping as well as positive reinterpretation and growth on the AL index. Furthermore, we investigated whether the AL index might be related to cognitive impairment observed in patients with psychosis (31, 33). We found that higher AL index is associated with worse performance of attention (in patients with psychosis and FHR-P individuals) and semantic fluency (in patients with psychosis). This association appeared to be non-significant in healthy controls. Interestingly, no significant correlations between the AL index and perceived stress or life stressors were found in the groups of patients and controls (30, 32, 33).

The association between the AL index and cognitive impairment is supported by the findings from studies investigating neurostructural correlates of the AL index in patients with schizophrenia (29, 34). Chiappelli et al. (29) found significantly lower average cortical thickness in patients with schizophrenia compared to controls. This difference appeared to be insignificant after accounting for the AL index. More specifically, higher diastolic blood pressure and heart rate as well as elevated levels of urine norepinephrine and CRP predicted reductions of cortical thickness in the group of patients. In healthy controls, only BMI and CRP levels were associated with cortical thickness. Another study examined the relationship between the AL index and white matter connectivity (29). The authors found that higher AL index was associated with lower fornix structural connectivity in patients with schizophrenia and healthy controls. There were also some correlation between the AL index and structural connectivity of internal capsule, corona radiata, thalamic radiation, sagittal striatum, and superior longitudinal fasciculus in patients with schizophrenia. However, these correlations were insignificant after correction for multiple testing.

## Summary of Evidence and Future Directions

Convincing evidence indicates systemic biological dysregulations associated with stress exposure in patients with psychotic disorders that occurs in the early course of illness. Elevated AL index might be related to more severe positive symptoms and functional impairment in psychosis. Although it has been reported that the AL index decreases in the course of antipsychotic treatment (27), it still remains elevated in patients with chronic schizophrenia (32, 33). The mechanisms of these alterations remain unknown. One scenario is that experiencing acute psychotic symptoms might itself trigger a number of biological responses. However, the AL index remains elevated in outpatients with schizophrenia (32). Our group revealed that decreased use of active coping strategies contribute to elevated AL index *via* the effects of depressive symptoms (30).

Less is known about the effects of stress on the AL index. Three studies revealed that recent stressors are unlikely to account for increased AL index in FEP patients and schizophrenia outpatients (30, 32, 33). Our group also found no association between lifetime stressors and the AL index in FEP patients (33). However, the list of threatening experiences (43) provides a limited insight into life stressors. A history of childhood trauma is a well-established risk factor of psychosis (5). Moreover, poor infant psychosocial environment has been associated with physiological dysregulations that can be conceptualized in frame of the AL concept (50). Therefore, investigating the effects of childhood trauma on the AL index serves as one of directions for future studies. Given that early-life stress is neither necessary nor sufficient to trigger the onset of psychosis as approximately one third of patients with psychosis report this type of experiences, insights into potential mediators and moderators should be taken into account (4, 6). Indeed, a number of psychological phenomena have been found to mediate the effects of traumatic events on psychosis risk, including cognitive biases and self disturbances (51, 52). Childhood adversities have been associated with increased AL index in adult non-clinical populations (53, 54); however, a mediating effect of social support has been reported (54). A lack of association between the measures of psychosocial stress and the AL index in previous studies of patients with psychosis might also imply that stress is not a primary mechanism of elevated AL index in this group of patients. Instead, physiological dysregulations captured by the AL index might result from processes that are inherent to the pathophysiology of psychosis. This scenario is supported by recent findings showing similar AL index in patients with FEP and FHR-P individuals (33). Moreover, reconsidering the definition of stressors might provide further insights into the mechanisms of elevated AL index in psychosis. Indeed, McEwen and Wingfield (24, 55) posited that other stressors, including infections, injuries or nutritional deficiencies may also contribute to allostatic overload.

Although a number of studies revealed increased AL index in patients with psychosis, causal inference cannot be established due to a cross-sectional design. However, indirect evidence suggests that stress response measured as the AL index might contribute to a number of neurostructural alterations that are typical for patients with schizophrenia, including reduced cortical thickness and white matter connectivity (29, 34). In agreement with these findings, we found that increased AL might account for working memory deficits (measured by the digit span task) observed in FEP patients (31). There were no significant correlations between working memory performance and the AL index in healthy controls. Working memory deficits are widely observed in patients with schizophrenia and bipolar disorder, and are more closely related to psychosis dimension than a diagnostic group itself (56, 57). Prefrontal cortex that represents one of main neural substrates of working memory contains a high density of glucocorticoid receptors (58). It has been shown that acute stress at high loads might impact working memory (59). In support of our results, a recent study by Reed et al. (60), revealed that the Trier Social Stress Test disrupted accuracy during the working memory task in chronic schizophrenia patients, but not in healthy controls. These findings suggest that patients with psychotic disorders and healthy controls might be differentially vulnerable to the effects of stress responses.

Investigating the AL concept in patients with psychotic disorders might also improve our understanding of the mechanisms underlying epigenetic alterations. Indeed, psychotic disorders are also characterized by a number of epigenetic alterations, especially differential DNA methylation, with some concordant patterns in the brain and peripheral tissues (61, 62). Our group also revealed that a history of childhood trauma might be associated with lower methylation of LINE-1 sequences, representing surrogate measures of global DNA methylation (14). There is also consistent evidence that a history of childhood trauma might be associated with global DNA hypomethylation and reduced expression of the *BDNF* gene in patients with psychosis (63). One of directions for future studies would be to test the mediating role of allostatic mechanisms in gene × environment interactions. One of candidate genes would be the *FKBP5*, encoding the FK506 binding protein, which acts as a co-chaperone for glucocorticoid receptors (64). Indeed, it has consistently been shown that variations in the *FKBP5* gene interact with early life stress and influence several outcomes of psychotic disorders [for review see (2)]. Moreover, allele-specific demethylation of the *FKBP5* gene has been found to mediate gene × childhood trauma interactions (65). Finally, a recent study by Lee et al. (66) demonstrated that blood *FKBP5* methylation and mRNA expression levels might be a marker of 30-day cortisol load.

It should be noted that there is no consensus regarding a calculation of the AL index and inclusion of specific markers. This inconsistency might explain differences regarding correlates of elevated AL index in patients with psychosis. To date, there are two main approaches of calculating the AL index—the one based on percentiles in healthy controls and the second one referring to conventional clinical thresholds. The use of thresholds established in the general population seems to be more relevant for clinical practice; however, it may underestimate subclinical physiological dysregulations widely observed at early stages of psychosis. Moreover, certain biomarkers, e.g., cytokines or BDNF, do not have established norms. In turn, the use of percentiles established in healthy controls, although widely implemented in previous studies of the AL concept, requires representative cohorts and appropriate matching of patients and healthy controls. Although several studies based on non-clinical populations revealed that the AL index better predicts morbidity and mortality, compared to traditional detection methods and single markers employed in biomedical practice (26), its clinical value for patients with psychosis remains unknown. At this point, it should be noted that the AL concept shares some similarity with other composite predictors, including metabolic syndrome and the Framingham Risk Score (67). However, it has been shown that the AL index is conceptually different and better predicts unfavorable health outcomes than metabolic syndrome (68, 69). This is particularly important since life expectancy is reduced up to 20 years in patients with psychosis, mostly due to high prevalence of metabolic syndrome and cardiovascular diseases (70, 71, 72). A consensus panel of AL mediators would enable to tailor early intervention aimed at reducing cardiovascular risk in psychotic disorders. In addition, repeated measures of the AL index would support monitoring the efficacy of pharmacotherapy and psychotherapy. Before implementation to clinical practice, longitudinal studies are required to investigate the effects of antipsychotics on the AL index. This is particularly important for antipsychotics that are known to be effective in terms of improving symptomatic outcomes but lead to the development of several metabolic side effects. Differentiating between the effects of antipsychotics on the AL index driven by their efficacy and those related to metabolic adversities is needed.

In summary, the AL concept holds a great promise for understanding the role of stress in the pathophysiology of psychotic disorders. Longitudinal studies of at risk populations, with a range of clinical data, are needed to disentangle dynamics and causal mechanisms of detrimental effects associated with stress exposure in psychosis. Resilience and vulnerability factors should be taken into account to understand differential effects of stress exposure in various populations. A consensus statement regarding the measurement of the AL in psychiatric research is needed to enable comparability and generalization of results from future studies as well as translation of predictive models into clinical practice.

## Author Contributions

BM confirms being the sole contributor of this work and has approved it for publication.

## Funding

This work was funded from science budget resources granted for the years 2016–2019 (the Iuventus Plus grant awarded by the Ministry of Science and Higher Education, grant number: IP2015 052474).

## Conflict of Interest

The author declares that the research was conducted in the absence of any commercial or financial relationships that could be construed as a potential conflict of interest.
